# Hydrocortisone Mitigates Alzheimer’s-Related Cognitive Decline through Modulating Oxidative Stress and Neuroinflammation

**DOI:** 10.3390/cells12192348

**Published:** 2023-09-25

**Authors:** Jinran Li, Long Chen, Sai Liu, Yuan Sun, Le Zhen, Zheying Zhu, Guangji Wang, Xinuo Li

**Affiliations:** 1 Jiangsu Provincial Key Laboratory of Drug Metabolism and Pharmacokinetics, China Pharmaceutical University, 639 Longmian Avenue, Nanjing 211166, China; 2 State Key Laboratory of Natural Medicines, China Pharmaceutical University, 639 Longmian Avenue, Nanjing 211166, China; 3 School of Pharmacy, The University of Nottingham, Nottingham NG7 2RD, UK

**Keywords:** Alzheimer’s disease, hydrocortisone, neuroinflammation, glial activation, oxidative stress

## Abstract

Alzheimer’s disease (AD), an age-related degenerative disorder, is characterized by β-amyloid deposition, abnormal phosphorylation of tau proteins, synaptic dysfunction, neuroinflammation, and oxidative stress. Despite extensive research, there are no medications or therapeutic interventions to completely treat and reverse AD. Herein, we explore the potential of hydrocortisone (HC), a natural and endogenous glucocorticoid known to have potent anti-inflammatory properties, in an Aβ_1–42_-induced AD mouse model. Our investigation highlights the beneficial effects of HC administration on cognitive impairment, synaptic function enhancement, and neuronal protection in Aβ_1–42_-induced AD mice. Notably, HC treatment effectively suppresses the hyperactivation of microglia and astrocytes, leading to a reduction in proinflammatory factors and alleviation of neuroinflammation. Furthermore, HC intervention demonstrates the capacity to mitigate the generation of ROS and oxidative stress. These compelling findings underscore the potential therapeutic application of HC in AD and present promising opportunities for its utilization in AD prevention and treatment. The implications drawn from our findings indicate that hydrocortisone holds promise as a viable candidate for adjunctive use with other anti-AD drugs for the clinical management of patients presenting with moderate to severe AD.

## 1. Introduction

Alzheimer’s disease (AD) is a progressive age-related neurodegenerative disease characterized by memory impairment, behavioral dysfunction, and emotional irritability [1]. Pathologically, AD is defined by β-amyloid deposition, abnormal phosphorylation of tau proteins, and the formation of neuronal fibrillary tangles (NFTs), ultimately resulting in synaptic dysfunction and neuronal death [2,3]. With an estimated global prevalence of 50 million patients, AD poses a significant threat to public health, overwhelmingly impacting affected patients, their families, and society at large [4,5,6]. Despite increasingly extensive research, the current treatment landscape remains inadequate, leaving a hugely unfulfilled need as no medications or interventions offer complete remission or reversal of AD. Consequently, the development of effective therapeutic strategies is of utmost urgency in addressing this formidable health challenge.

AD is a complex neurodegenerative condition with an uncertain pathogenesis. Numerous mechanistic hypotheses, including the amyloid hypothesis, tau hypothesis, inflammation hypothesis, and cholinergic hypothesis, have been proposed to explain the disease’s underlying mechanisms [7,8,9,10,11,12]. As research has progressed, neuroinflammation has emerged as a key pathological feature alongside beta-amyloid plaques and abnormal tau phosphorylation. In AD patients, the presence of inflammatory factors and acute response proteins, such as IL-6 and TNF-α, have been reported in the brain [13,14,15]. Notably, microglia and astrocytes play central roles in mediating neuroinflammation. Microglia, as key components of the brain’s innate immune system, initially engage in defense through Aβ phagocytosis during the early stages of AD [16]. However, under conditions of persistent chronic activation by Aβ, microglia aggravate neuroinflammation by releasing pro-inflammatory factors and cytotoxic substances, ultimately leading to neuronal dysfunction and amyloid deposition [13,17]. Similarly, astrocytes, typically supportive of neuronal survival and synaptic functions, undergo reactive transformations in the AD pathological state, releasing neurotoxic factors and affecting calcium homeostasis and intracellular and extracellular stability, ultimately causing Aβ plaque accumulation, synapse loss, and an inflammatory response [18,19,20]. Microglia and astrocytes play pivotal roles in mediating neuroinflammation, contributing to neuronal dysfunction and amyloid deposition. Targeting microglial and astrocytic hyperactivation offers a potential therapeutic avenue for AD intervention [21,22,23].

In addition to neuroinflammation, oxidative stress constitutes a significant factor closely linked to AD [24]. Previous studies have shown that Aβ plaque deposition, mitochondrial dysfunction, and inflammation contribute to the induction of oxidative stress [25]. Upon encountering oxidative stress, neurons incur damage, interacting with reactive oxygen species (ROS), leading to synaptic dysfunction and neuronal cell apoptosis, thereby exacerbating the AD pathological process [26,27]. In conclusion, targeting inflammation and mitigating oxidative stress damage emerge as promising approaches for preventing and treating AD.

Glucocorticoids (GCs) are widely used as anti-inflammatory agents, and their combination with other drugs has demonstrated potential advantages and neuroprotective effects [28,29]. Additionally, they have shown valuable efficacy in ameliorating the pathological features of AD and exerting protective and regulatory roles [30,31]. Among the natural and endogenous GCs, hydrocortisone (HC) stands out for its potent anti-inflammatory effects, yielding unique pharmacological benefits in autoimmune disease, septic shock, and allergic conditions [32,33]. HC exerts neuroprotection by restraining cytotoxic substances released by activated microglia and impeding astrocyte proliferation [34,35].

HC has garnered considerable attention due to its acknowledged anti-inflammatory and neuroprotective properties and its regulatory role in stress conditions, prompting further exploration of its potential therapeutic implications for AD. Although initial evidence suggests a potential association between plasma hydrocortisone levels and reduced AD incidence, a comprehensive understanding of HC’s specific role in AD pathogenesis remains elusive [36]. This study aims to provide an in-depth investigation into the therapeutic potential of HC for AD, offering valuable insights for its future application in combination with other anti-AD drugs and potentially paving the way for novel medication approaches in clinical AD management.

## 2. Materials and Methods

### 2.1. Systematic Review and Meta-Analysis

A systematic review and meta-analysis followed the PRISMA (Preferred Reporting Items for Systematic Reviews and Meta-Analyses) statement guidelines [37]. We systematically searched PubMed, Cochrane, MEDLINE, and Web of Science in July 2023 and additionally screened the references of the included articles.

Studies that assessed the associations of the anti-inflammation agent and incident dementia or AD were included and the name of the first author, publication year, cohort name, country, number of participants, age range at baseline, dietary assessment methods, dementia ascertainment methods, and risk estimates and 95% CIs from the multivariable adjusted models and included covariates were extracted. The Newcastle–Ottawa scale was used to assess the quality of the included studies. SL and LC independently screened the literature, extracted the data, and assessed the risk of bias. Disagreement and discordance were resolved by the third author JL.

Common-effect models were used to pool the risk estimates comparing the anti-inflammation agent group and placebo group with the reported I^2^ statistic using R (v 4.3.1) package meta (R Project for Statistical Computing).

### 2.2. Cell Culture

The SH-SY5Y cell line (Shanghai Zhongqiaoxinzhou Company, Shanghai, China) was cultured in Dulbecco’s modified Eagle medium (DMEM, Invitrogen, Carlsbad, CA, USA) supplemented with 10% FBS, 100 IU/mL penicillin, and 100 ug/mL streptomycin in a humidified atmosphere of 5% CO_2_ at 37 °C.

### 2.3. Protective Effect of HC on Aβ_1–42_-Injured SH-SY5Y Cells

The SH-SY5Y cells were seeded into 96-well plates and incubated overnight until they reached about 70% confluence. The cells in the control group were cultured in DMEM for 36 h, and the model group were cultured with DMEM for 12 h and treated with 10 μM Aβ_1–42_ for 24 h. In the HC treatment group, cells were pretreated with 0.5, 1, and 2 μM HC for 12 h, and then treated with 10 μM Aβ_1–42_ for 24 h. Finally, the cell viabilities were detected using the MTT method and the absorbance was measured with a microplate reader (Molecular Devices, SpectraMax Mini, San Jose, CA, USA).

### 2.4. Animals and Drug Administration

All male C57/BL6 mice (6 weeks old, weight 18–22 g) were purchased from SpePharm Biotechnology Company (Beijing, China). The animal culture and procedures were approved by the Pharmaceutical Laboratory Animal Center of China Pharmaceutical University. Recombinant human Aβ_1–42_ peptide (Beyotime Biotechnology, Shanghai, China) was dissolved in sterile PBS at a concentration of 2 mg/mL and incubated for 24 h at 37 °C to obtain aggregated Aβ_1–42_. All the mice were randomly divided into three groups (*n* = 8 per group): the control group, the Aβ_1–42_ (intracerebroventricular injection, i.c.v.) group, and the Aβ_1–42_ (intracerebroventricular injection, i.c.v.) + HC (25 mg/kg, intragastric administration, i.g.) group. The control group was injected with 5 uL saline and the other mice were injected with 5 uL aggregated Aβ_1–42_ into the lateral ventricle through the brain stereo-positioning instrument (Harvard). HC (dissolved in saline) was intragastrically administrated after surgery for 14 days. The mice in the model and control group were gavaged with only normal saline.

### 2.5. Morris Water Maze (MWM)

During days 9–14, the Morris water maze test was performed for cognitive function measurement [38]. The MWM system mainly consisted of a black circular pool, a circular hidden platform which was submerged 1 cm below the water surface, and a video analysis system. The experimental maze was divided into four quadrants and the target platform was placed in the middle of the third quadrant. The experiment lasted 6 days and was conducted in two stages, including the hidden platform training for 5 consecutive days and the probe trial on day 6. In the hidden platform training, the mice were allowed to swim for 90 s to find the target platform which was 1 cm above the water. Each mouse entered the pool from the same position which was opposite the third quadrant. The video analysis system automatically recorded the time when each mouse climbed onto the platform. If the mouse failed to reach the platform within 90 s, it was manually guided to the target and left to rest on the platform for 10 s to remember the position. The probe trial was carried out on the 6th day, and the latency, path length, swimming velocity, target quadrant residence time, and traveled trajectory were recorded and analyzed through the video tracking system.

### 2.6. RNA Extraction and Real-Time PCR

The mice under deep anesthesia were perfused with frozen PBS (PH = 7.4) transcardially and the brains were collected and stored at −20 °C. The total RNA was purified from the cerebral cortex with Trizol Reagent (Vazyme, Nanjing, China) and reversed-transcribed to cDNA with HiScript III RT SuperMix (Vazyme, Nanjing, China) according to the manufacturer’s instructions. The cDNA samples were amplified using the CFX Opus Real-Time PCR System (Bio-Rad, v2.2) using Taq Pro Universal SYBR qPCR Master Mix (Vazyme, Nanjing, China). Relative expression changes were analyzed using the 2^−ΔΔCt^ method and the target gene expression levels were normalized to GAPDH. The specific primer sequences for RT-PCR are listed in Supplementary Table S1.

### 2.7. Immunofluorescence

Immunofluorescence was performed as described previously [39]. The mice were perfused with frozen PBS (PH = 7.4) transcardially and then perfused with 4% paraformaldehyde (PFA) for tissue fixation. After cardiac perfusion, the brains were harvested, fixed in 4% PFA for at least 24 h and protected from light at 4 °C. The fixed brain tissue was transferred into a 30% sucrose solution for dehydration. After dehydration for 48 h, the brains were cut into 25 μm thick coronal slices using a freezing microtome (Leica, Wetzlar, Germany, CM1950), and the slices with an intact hippocampus were stored in the freezing solution (PBS:ethylene glycol:glycerin = 5:3:2) and stored at −20 °C.

The brain slices were washed 3 times in PBS, followed by treatment in 0.3% Triton X-100 (Beyotime Biotechnology, Shanghai, China, ST795) for 20 min at room temperature. After being blocked with 5% bovine serum albumin (Beyotime, Shanghai, China, ST023) diluted with PBS for 1 h at room temperature, the slices were incubated with the primary antibody (rabbit anti-PSD95 (Abcam, Cambridge, UK, ab18258, 1:200), rabbit anti-iBA1 (Fujifilm, Tokyo, Japan, 019-19741, 1:300), and rabbit anti-GFAP (CST, Boston, MA, USA 80788, 1:200)) overnight at 4 °C. Then the secondary antibody (goat anti-rabbit IgG H&L (Alexa Fluor^®^ 488) (Abcam, Cambridge, UK, ab150077, 1:500)) was applied for 1 h at room temperature, and the slices were finally counterstained with 1 μg/mL DAPI (Beyotime Biotechnology, Shanghai, China, C1002) for 20 min. After being washed with PBS, the samples were visualized with a fluorescence microscope (BioTek, Hong Kong, China, Cytation5), and images were collected with a MicroImaging System (BioTek, Hong Kong, China, Cytation5). Finally, the ImageJ software (v1.54f) was used to analyze and quantify acquired images.

### 2.8. Determination of ROS Levels

SH-SY5Y cells were seeded into 24-well plates and incubated overnight. After modeling and hydrocortisone intervention, the cells were incubated with a 10 μM dihydroethidium (DHE) (Beyotime, Shanghai, China) solution for 30 min at 37 °C, followed by rinsing with PBS 3 times. The brain sections were subjected to a series of prepared protocols. The frozen brain sections were also incubated with 10 μM DHE for 30 min at 37 °C. The images of cells and brains were captured using a microscope (BioTek, Hong Kong, China, Cytation5) and analyzed with Image J software (v1.54f).

### 2.9. Western Blot Analyses

Western blot analyses was performed as described previously [31]. Cerebral cortex tissues were lysed with RIPA buffer (Beyotime, Shanghai, China) supplemented with phenylmethanesulfonyl fluoride (PMSF). After centrifugation at 12,000 rpm/min at 4 °C for 30 min, the supernatant was collected and a BCA kit (Beyotime, Hong Kong, China) was used for protein quantification. The protein was isolated by sodium dodecyl sulfate–polyacrylamide gel electrophoresis (SDS-PAGE) and transferred onto polyvinylidene difluoride fluoride (PVDF) membranes (Bio-Rad, Hercules, CA, USA). After blocking with 5% skim milk for 1 h at room temperature, the membranes were incubated with the primary antibody (Phospho-NF-kB p65, AffinitY, AF2006, 1:1000) overnight at 4 °C. After washing with TBST 5 times, the membranes were then incubated with the secondary antibodies (HRP-conjugated anti-rabbit IgG) for 1 h. Protein expression was detected with an enhanced chemiluminescence (ECL) method with a gel imaging system (Bio-Rad, ChemiDoc MP, USA). Densities were normalized to GAPDH intensity levels and ImageJ software (v1.54f) was used for quantification.

### 2.10. Statistical Analyses

All the histograms and line charts were made with GraphPad Prism 8.0. The results are expressed as means ± SEM. At least three biological replicates were performed for all experiments to ensure consistency. Significant differences between two different groups were obtained using an unpaired Student’s *t*-test. A one-way ANOVA and the original FDR method of Benjamini and Hochberg were used to compare multiple independent groups. Statistical significant differences are indicated as * *p*-value < 0.05.

## 3. Results

### 3.1. Meta-Analysis in AD Patients

**Figure 1:**
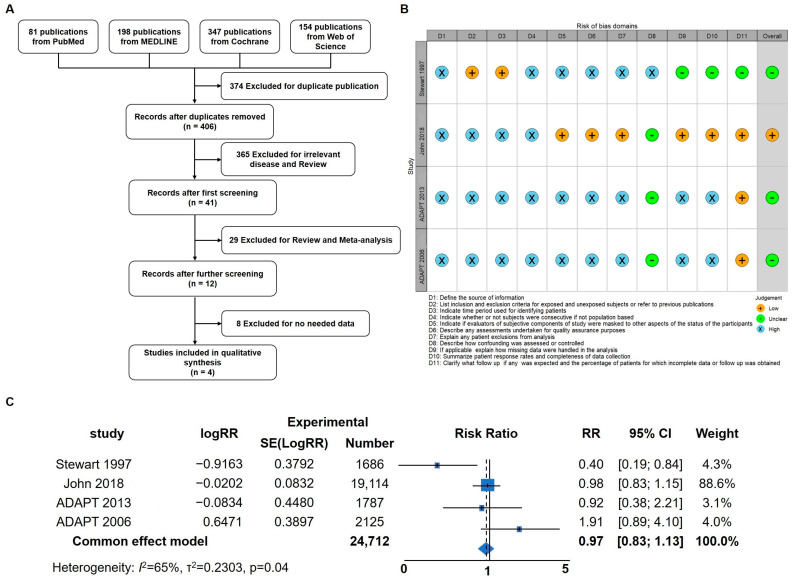
Meta-Analysis indicating that anti-inflammatory strategies could benefit AD patients. (**A**) Flow diagram of the four studies included in the meta-analysis. (**B**) Risk of bias summary: review authors’ judgements about each risk of bias item for each included study. (**C**) Forest plot of pooled risk ratio (RR) for the anti-inflammation agent on the prevention or treatment of AD using the random effects model [37,40,41,42].

Inflammation has been identified as a critical factor in the pathogenesis of Alzheimer’s disease (AD), and previous studies have demonstrated that anti-inflammatory treatments targeting neuroinflammation can ameliorate AD symptoms in mice [31]. To delve deeper into the impact of anti-inflammatory treatment strategies on AD symptoms in human patients, a meta-analysis was conducted to assess the potential benefits of anti-inflammatory interventions. Initially, a total of 780 articles were identified, of which 374 duplicates were removed. Following title and abstract screening, 394 articles were excluded, leaving 12 for full-text screening. Ultimately, four articles were included for the meta-analysis (Figure 1A,B) [37,40,41,42]. The results of the meta-analysis revealed that anti-inflammatory strategies may offer a certain degree of relief for AD patients (RR; 95% confidence interval [CI], 0.98 to 1.13; *p* = 0.04). Considering the prominent anti-inflammatory effects of hydrocortisone (HC), this prompted further investigation into its potential as a drug candidate for AD intervention and treatment. These findings underscore the importance of anti-inflammatory approaches in AD management and provide a basis for exploring HC as a potential therapeutic agent for AD in future research.

### 3.2. Behavioral and Cognitive Function In Vivo

**Figure 2:**
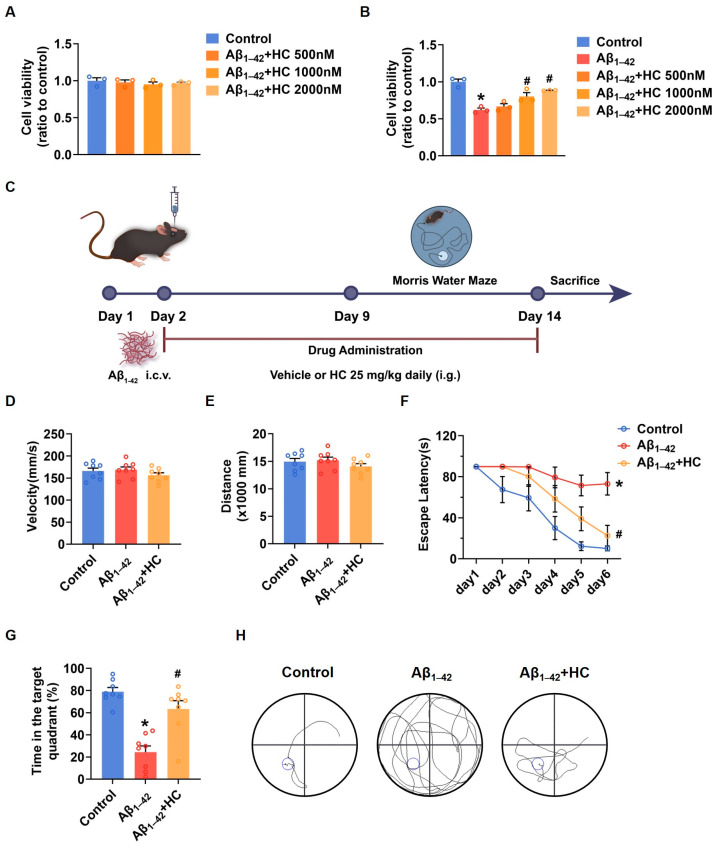
HC attenuated the injury induced by Aβ_1–42_ in vitro and improved the behavioral and cognitive function in vivo. (**A**) Cytotoxicity of HC on SH-SY5Y cells. (**B**) Effects of HC on the cell viability of SH-SY5Y cells induced by Aβ_1–42_. (**C**) Scheme of experimental design. (**D**) The average swimming velocity among all groups on the first day. (**E**) The average swimming distance among all groups on the first day. (**F**) The latency to locate the target platform during the experiment. (**G**) Time spent in the target quadrant in the probe test. (**H**) The swimming paths of mice finding the hidden platform in the probe test. Data are shown as mean ± SEM. * *p* < 0.05 vs. control group; ^#^ *p* < 0.05 vs. Aβ_1–42_-induced group.

To gain insights into the potential of HC as a potent anti-inflammatory drug in AD treatment, we initiated investigations of its effects in SH-SY5Y cells. The MTT results demonstrated that HC showed no significant cytotoxicity at concentrations of 500, 1000, or 2000 nmol/L, as cell viability remained unchanged within this range (Figure 2A). When SH-SY5Y cells were exposed to Aβ_1–42_, the cell viability significantly decreased compared to the control group. However, pretreatment with HC at various concentrations for 12 h increased cell viability, indicating a dose-dependent protective effect of HC against Aβ_1–42_-induced damage (Figure 2B).

Furthermore, to confirm the protective effect of HC in AD, we evaluated its impact on cognitive function in Aβ_1–42_-induced mice using the Morris water maze (MWM) behavioral test (Figure 2C). Both control and Aβ_1–42_-induced mice were orally administered either a vehicle solution or HC for two weeks, followed by behavioral testing for one week. To ensure that the mice did not exhibit motor impairments during the test, we measured average swimming speed and distance traveled on the first day (Figure 2D,E), which confirmed the absence of movement disorders in all mice. Notably, the Aβ_1–42_-induced mice displayed longer times to locate the hidden platform compared to the control mice, indicative of lower memory levels. In contrast, the HC-treated Aβ_1–42_-induced mice exhibited improved performance, finding the hidden platform faster than the Aβ_1–42_-induced mice (Figure 2F).

Moreover, the HC-treated Aβ_1–42_-induced mice spent more time in the hidden platform quadrant in the probe trial than the Aβ_1–42_-induced mice (Figure 2G). The HC-treated group also demonstrated a more linear and targeted search strategy compared to the Aβ_1–42_-induced mice (Figure 2H). Overall, these results indicate that HC ameliorated Aβ_1–42_-induced cell damage and enhanced the behavioral and cognitive functions of the Aβ_1–42_-induced mice, suggesting the potential beneficial role of HC in AD.

### 3.3. Synaptic Dysfunction in Aβ_1–42_-Induced Mice

**Figure 3:**
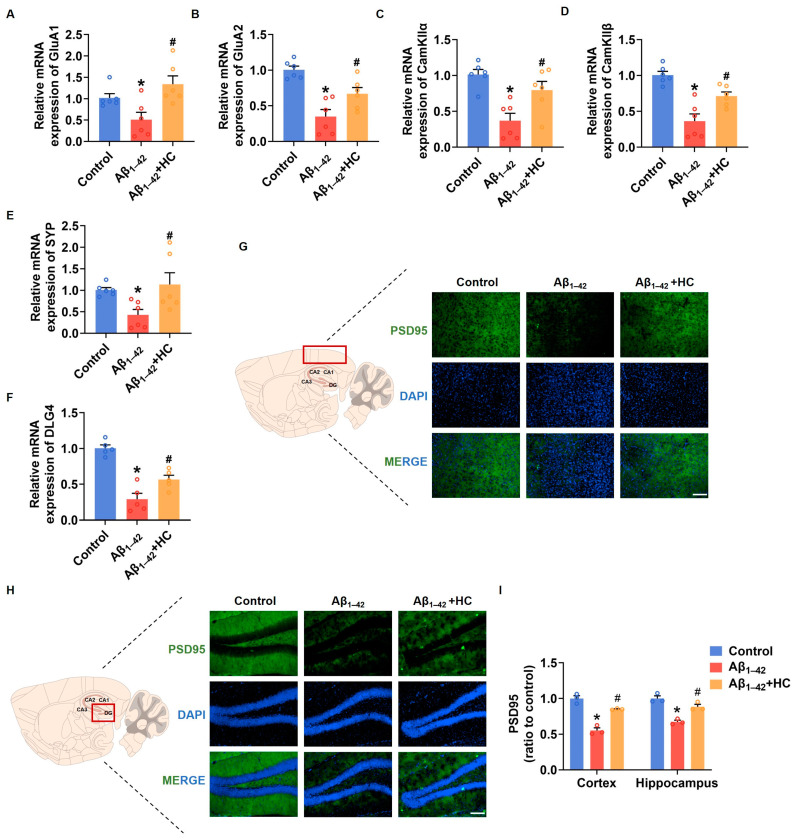
HC-alleviated synaptic dysfunction in Aβ_1–42_-induced mice. (**A**) The relative mRNA levels of GluA1 in the cerebral cortexes of mice. (**B**) The relative mRNA levels of GluA2 in the cerebral cortexes of mice. (**C**) The relative mRNA levels of CamKIIα in the cerebral cortexes of mice. (**D**) The relative mRNA levels of CamKIIβ in the cerebral cortexes of mice. (**E**) The relative mRNA levels of Synaptophysin in the cerebral cortexes of mice. (**F**) The relative mRNA levels of Dlg4 in the cerebral cortexes of mice. (**G**) Representative fluorescence micrographs showing PSD95 expression in the cerebral cortexes (Scale bar, 200 μm). (**H**) Representative fluorescence micrographs showing PSD95 expression in the hippocampi (Scale bar, 200 μm). (**I**) Quantification of the total number of PSD95^+^ cells in the cerebral cortexes and hippocampi. Data are shown as mean ± SEM. * *p* < 0.05 vs. control group; ^#^ *p* < 0.05 vs. Aβ_1–42_-induced group.

Synaptic damage represents a prominent pathological feature of AD. The accumulation of Aβ and the subsequent increase in free radicals contribute to synaptic loss and damage, ultimately leading to neuronal impairment and cognitive deficits [43]. In this context, enhancing synaptic density and improving synaptic function have emerged as potential strategies to prevent and ameliorate cognitive impairment in AD [44]. To investigate the potential benefits of HC in alleviating synaptic damage, we examined the mRNA expression levels of synaptic-function-related factors in the cortexes of mice brains. Notably, the mRNA expression of GluA1 and GluA2, two subunits of the α-amino-3-hydroxy-5-methyl-4-isoxazolepropionic acid (AMPA) receptor involved in neural signaling; CaM-dependent protein kinase IIα (CamKIIα); CaM-dependent protein kinase IIβ (CamKIIβ), critical for synaptic plasticity; and synaptophysin (SYP), a synaptic vesicle protein associated with synaptic remodeling, were significantly down-regulated in the Aβ_1–42_-induced mice. However, HC treatment significantly increased the mRNA expression of GluA1, GluA2, CaMKIIα, CamKIIβ, and SYP in vivo [45,46,47]. Moreover, postsynaptic density protein-95 (PSD95), encoded by Dlg4, indicates synaptic function [48]. The HC treatment substantially increased the mRNA expression level of Dlg4, and the immunofluorescence assay indicated that the expression levels of PSD95 were also significantly increased in the cerebral cortexes and hippocampi of mice following the HC treatment (Figure 3F,G). These observations and data underscore the critical role of HC in repairing synaptic dysfunction and enhancing synaptic function, pointing towards its potential therapeutic significance in AD treatment. The beneficial effects of HC on synaptic integrity may hold promise for promoting cognitive improvements and ultimately contributing to the management of AD pathology.

### 3.4. The Inflammatory Responses in Aβ_1–42_-Induced Mice

**Figure 4:**
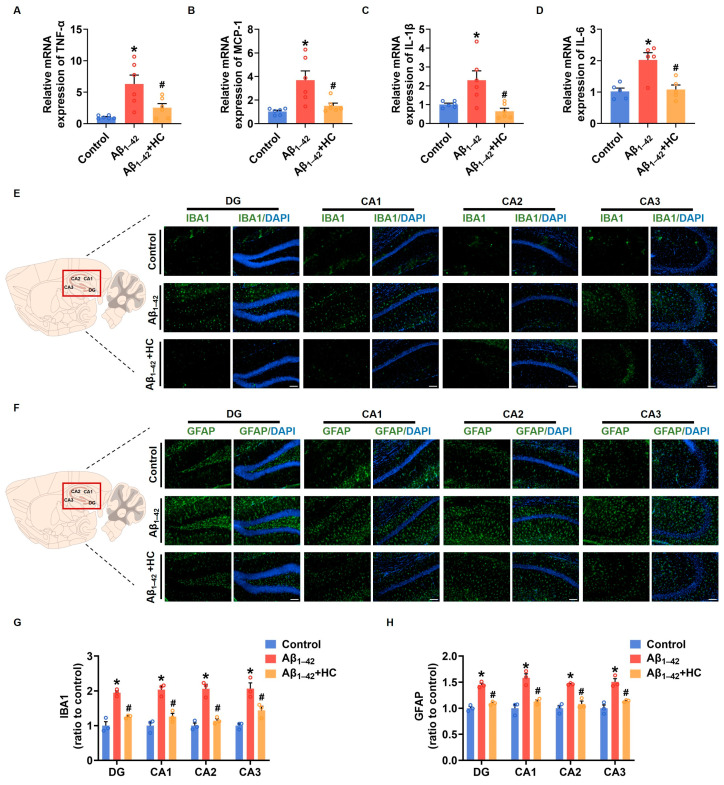
HC attenuated the inflammatory responses in Aβ_1–42_-induced mice. (**A**) The relative mRNA levels of TNF-α in the cerebral cortexes of mice. (**B**) The relative mRNA levels of MCP-1 in the cerebral cortexes of mice. (**C**) The relative mRNA levels of IL-1β in the cerebral cortexes of mice. (**D**) The relative mRNA levels of IL-6 in the cerebral cortexes of mice. (**E**) Representative fluorescence micrographs showing IBA1 expression in the DG, CA1, CA2, and CA3 of hippocampi (scale bar, 200 μm). (**F**) Representative fluorescence micrographs showing GFAP expression in the DG, CA1, CA2, and CA3 of hippocampi (Scale bar, 200 μm). (**G**) Quantification of the total number of IBA1^+^ cells in the DG, CA1, CA2, and CA3 of hippocampi. (**H**) Quantification of the total number of GFAP^+^ cells in the DG, CA1, CA2, and CA3 of hippocampi. Data are shown as mean ± SEM. * *p* < 0.05 vs. control group; ^#^ *p* < 0.05 vs. Aβ_1–42_-induced group.

Neuroinflammation mediated by microglia and reactive astrocytes plays a significant role in promoting the pathological process of AD, leading to synaptic dysfunction and cognitive impairment [49]. In order to evaluate the anti-inflammatory effect of HC in Aβ_1–42_-induced mice, we examined the mRNA levels of various proinflammatory factors. Results from the RT-PCR assay demonstrated that the HC treatment significantly decreased mRNA levels of pro-inflammatory factors, including TNF-α, MCP-1, IL-1β, and IL-6 when compared to the Aβ_1–42_-induced mice (Figure 4A–D). The immunofluorescence analysis of IBA1 and GFAP, markers for microglia and reactive astrocytes, respectively, revealed that Aβ_1–42_ induced excessive activation of microglia and astrocytes in different hippocampal regions such as DG, CA1, CA2, and CA3. However, the HC intervention effectively reduced the number of activated microglia and astrocytes (Figure 4E–H). Additionally, NF-κB activation, an inflammatory transcription factor, plays a crucial role in neuroinflammation and the accumulation of Aβ plaques, contributing to AD pathogenesis [50]. Our results indicated that the HC treatment effectively blocked the Aβ_1–42_ induction’s function in increasing protein levels of phosphorylated NF-κB (Figure S1). Collectively, these data strongly suggest that HC may prevent the activation of microglia and astrocytes, exerting anti-inflammatory effects, and thereby safeguarding synaptic function and ameliorating cognitive impairment. These findings highlight the potential of HC as a therapeutic agent to target neuroinflammation in AD and underscore its significance in modulating the intricate pathogenesis of the disease.

### 3.5. Oxidative Stress and Neuroprotection

**Figure 5:**
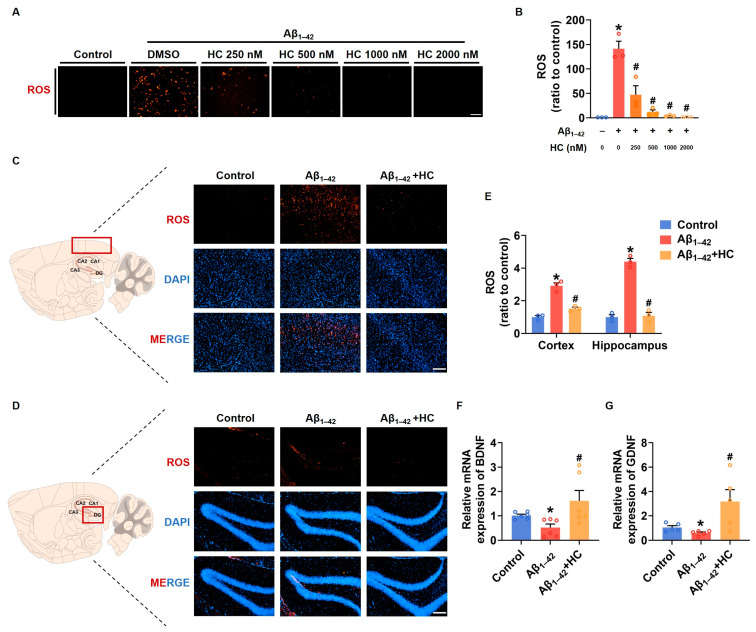
HC-reversed oxidative stress and exerted neuroprotective effect. (**A**) Representative images of DHE staining in SH-SY5Y cells (scale bar, 200 μm). (**B**) Quantification of relative ROS levels in SH-SY5Y cells. (**C**) Representative fluorescence micrographs showing ROS levels in the cerebral cortexes (scale bar, 200 μm). (**D**) Representative fluorescence micrographs showing ROS levels in the hippocampi (scale bar, 200 μm). (**E**) Quantification of relative ROS levels in the cerebral cortexes and hippocampi. (**F**) The relative mRNA levels of BDNF in the cerebral cortexes of mice. (**G**) The relative mRNA levels of GDNF in the cerebral cortexes of mice. Data are shown as mean ± SEM. * *p* < 0.05 vs. control group; ^#^ *p* < 0.05 vs. Aβ_1–42_-induced group.

Similar to neuroinflammation, multi-factor-induced oxidative stress is also considered a core pathogenesis of AD [26]. Chronic oxidative stress can lead to impaired nerve cell function and an enhanced inflammatory response [51]. To investigate the potential role of HC in ameliorating oxidative stress, we examined the expression of ROS after HC treatment both in vitro and in vivo. Our findings demonstrated that the ROS levels in SH-SY5Y cells significantly increased after Aβ_1–42_ induction, and the HC treatment effectively reduced the ROS levels in a dose-dependent manner (Figure 5A,B). Similarly, the ROS fluorescent signal was also diminished in the hippocampi and cortexes of the HC-treated Aβ_1–42_-induced mice, indicating less oxidative stress injury (Figure 5C–E). Furthermore, to examine the neuroprotective effect of HC, we assessed the mRNA levels of the neurotrophic factors BDNF and GDNF, which promote neuronal survival and repair neuronal damage. Following the HC treatment, the mRNA levels of BDNF and GDNF were significantly increased (Figure 5F,G), further supporting the benefits of HC in inhibiting oxidative stress and protecting neurons.

**Figure 6:**
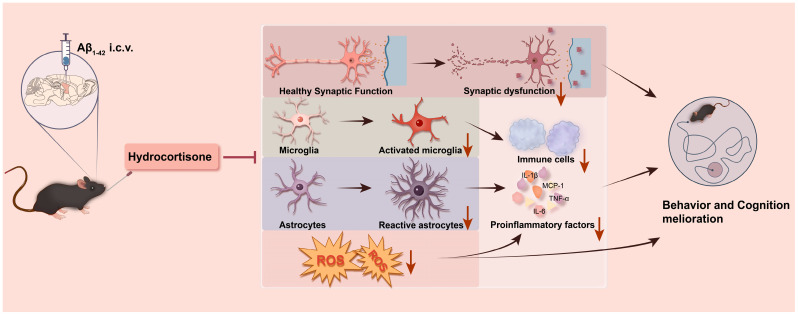
A summary graph of HC ameliorating learning and cognitive dysfunction in Aβ_1–42_-induced mice. HC may improve the behavior and learning dysfunction of Aβ_1–42_-induced mice through synaptic enhancement and neuroprotective functions. Meanwhile, HC inhibits the activation of microglia and astrocytes and reduces the level of ROS, thereby hindering the release of pro-inflammatory cytokines, resulting in cognitive function improvement.

Our results demonstrate that HC treatment can ameliorate synaptic dysfunction and protect neurons by inhibiting neuroinflammation and oxidative stress, ultimately leading to improved learning and cognitive function in Aβ_1–42_-induced mice (Figure 6). These findings highlight the potential therapeutic value of HC in mitigating the multifaceted pathogenesis of AD and offer promising avenues for future research and clinical intervention.

## 4. Discussion

Alzheimer’s disease (AD) remains a significant global public health challenge, with its pathogenesis attributed to various factors, including Aβ senile plaque deposition, hyperphosphorylation of tau proteins, and neuroinflammation [52]. However, the existing medications for AD merely offer temporary symptomatic relief without achieving a complete reversal of the underlying pathological processes. Notably, the failure of single-targeted anti-AD drugs has led to a shift towards exploring multi-targeted drugs or drug combinations as crucial alternatives for AD treatment. Particularly, the combination of hormonal anti-inflammatory drugs with other therapeutic agents shows promising potential as a treatment option for future clinical AD patients. Several therapeutic approaches have been developed, focusing on the inhibition of activated microglia and astrocytes, which significantly contribute to the exacerbation of neuroinflammation [53]. Our meta-analysis reinforces the significance of anti-inflammatory drugs as a promising avenue for AD treatment. In conclusion, the pursuit of multi-targeted therapeutic strategies and combinations may offer new hope in effectively addressing the complexities of AD pathogenesis and improving patient outcomes in the future.

HC, an essential natural glucocorticoid and potent anti-inflammatory agent, holds considerable significance in various conditions, including rheumatoid arthritis, allergic diseases, severe infections, and shock. Moreover, HC is a suitable candidate for inhibiting inflammatory responses within the brain. Studies have demonstrated the beneficial impact of HC on learning and memory function and its potential application in treating central nervous system injury by targeting microglia and astrocytes has been explored [54]. Despite these promising findings, the specific therapeutic effect of HC on AD remains uncertain and necessitates further investigation. As a potential avenue for AD treatment, understanding HC’s role in disease pathology and its interactions with neuroinflammatory processes may pave the way for novel therapeutic strategies in managing AD and improving cognition.

Our results provide support for the neuroprotective effect of HC in vitro, as demonstrated by its non-cytotoxic nature over a wide concentration range and its ability to alleviate Aβ_1–42_-induced injury in SH-SY5Y cells. Our investigation further revealed that HC exerted a considerable inhibitory effect on the pro-inflammatory cytokines induced by Aβ_1–42_ in mice. The immunofluorescence assay confirmed HC’s ability to impede the activation of microglia and astrocytes, which play critical roles in neuroinflammation. Our findings revealed HC’s ability to attenuate phosphorylated NF-κB protein levels (Figure S1). The NF-κB signaling pathway has been recognized as a compelling therapeutic target for AD, given its critical role in promoting microglial and astrocytic activation and consequent neuroinflammation [39,55,56]. The accumulation of Aβ triggers the activation of NF-κB, instigating an inflammatory response, while pro-inflammatory factors further increase NF-κB levels, creating a detrimental cycle. In light of these observations, our study provides valuable insights into HC’s potential role in AD pathogenesis by inhibiting microglia and astrocyte activation via the NF-κB signaling pathway, ultimately modulating neuroinflammation. These findings hold promise for advancing our understanding of HC’s therapeutic potential in mitigating AD-associated neuroinflammatory processes and offer potential avenues for future therapeutic interventions.

Oxidative stress is a pivotal factor in the pathogenesis of Alzheimer’s disease (AD), as it plays a critical role in bridging multiple pathways [26]. Reactive oxygen species (ROS) serve as a prominent characteristic of oxidative stress, exerting detrimental effects on synaptic function and neurotransmission, thereby leading to neurodegeneration and cognitive impairment [57]. Additionally, ROS further triggers microglia and astrocytes to release pro-inflammatory factors, promoting neuroinflammation, which reciprocally amplifies ROS levels [58]. The interplay between ROS and neuroinflammation results in neuronal dysfunction and cell death. Consequently, reducing ROS levels represents a critical avenue for intervening in AD and has garnered extensive investigation. In our study, ROS levels were assessed in Aβ_1–42_-induced SH-SY5Y cells in vitro and in an acute AD mouse model, revealing a decrease in ROS levels following the HC treatment. Furthermore, the HC treatment was observed to significantly increase the levels of neurotrophic factors and synapse function-related proteins. These findings suggest that HC may confer neuroprotection by reducing ROS levels, thereby mitigating oxidative stress-induced injury and presenting a potential therapeutic strategy for AD treatment. The modulation of ROS levels and its interaction with neuroinflammatory processes through HC administration hold significant implications for advancing AD research and fostering the development of novel therapeutic interventions.

The complexity and diversity of the pathological mechanisms involved in Alzheimer’s disease (AD) have highlighted the limitations of single-target medications in meeting therapeutic needs. Consequently, there has been an increasing emphasis on multi-target and combinatorial medications as promising approaches. Notably, the combination of memantine with cholinesterase inhibitors like donepezil and galantamine has gained widespread recognition for its substantial cognitive benefits [59,60]. Additionally, the combination of dexamethasone with acyclovir, an anti-herpes virus drug, has demonstrated efficacy in improving cognitive impairment induced by Aβ_1–42_ in mice [30]. Given these promising findings, the prospect of combining lower hydrocortisone (HC) doses with anti-AD drugs, such as donepezil, emerges as a viable approach. This combination reduces the required dose of donepezil and acts as a synergistic agent, potentially mitigating adverse effects and reducing overall toxicity.

While our study has yielded promising results regarding the neuroprotective effects of hydrocortisone (HC) in inhibiting neuroinflammation and oxidative stress, important limitations warrant further investigation. Notably, several studies have indicated that endogenous HC levels are elevated in the brains of AD patients, and excessive HC doses have been linked to hippocampal toxicity [61]. Considering the complex effects of HC, our current study has solely examined the high-dose effects of HC on cells, necessitating additional in vivo data to substantiate these findings. Our research has been limited to cell and animal models, warranting further exploration through clinical studies to establish robust references for future applications.

## 5. Conclusions

Our study underscores the potential of HC in protecting synapses and neurons through its capacity to inhibit neuroinflammation and oxidative stress, leading to improvements in cognitive and learning deficits in Aβ_1–42_-induced mice. These findings suggest that HC may hold promise in AD applications and provide encouraging prospects for future preventive and therapeutic approaches in AD management. By addressing the limitations and exploring the translational potential of HC in clinical settings, we can advance our understanding of its therapeutic efficacy and optimize its usage for the benefit of AD patients. The combined efforts of further research and clinical evaluation will pave the way for potential AD prevention and treatment strategy advancements.

## Data Availability

The original contributions presented in the study are included in the article/Supplementary Material. Further inquiries can be directed to the corresponding authors.

## Supplementary Materials

The following supporting information can be downloaded at: https://www.mdpi.com/article/10.3390/cells12192348/s1, Figure S1: Effects of HC on the expression levels of p-NF-κB in Aβ_1–42_-induced mice; Table S1: The primer sequences for RT-PCR.
