# Supplementary figures and images for: Hydrocortisone Mitigates Alzheimer’s-Related Cognitive Decline through Modulating Oxidative Stress and Neuroinflammation
Source: Cells. 2023 Sep 25;12(19):2348. doi: 10.3390/cells12192348 (PMC10571890; doi:10.3390/cells12192348)

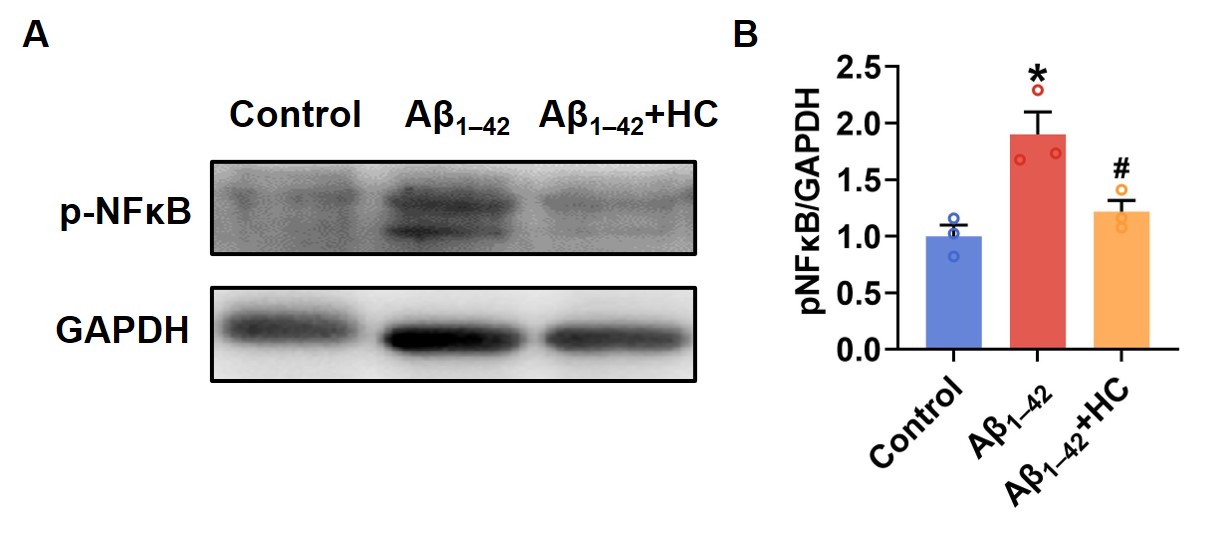

Supplement: Supplementary file 1 [file cells-12-02348-s001.zip › Supplementary Files/Supplementary Figure 1.jpg]
